# Mitochondrial oxidative stress is the achille's heel of melanoma cells resistant to Braf-mutant inhibitor

**DOI:** 10.18632/oncotarget.1420

**Published:** 2013-10-06

**Authors:** Paola Corazao-Rozas, Pierre Guerreschi, Manel Jendoubi, Fanny André, Aurélie Jonneaux, Camille Scalbert, Guillaume Garçon, Myriam Malet-Martino, Stéphane Balayssac, Stephane Rocchi, Ariel Savina, Pierre Formstecher, Laurent Mortier, Jérome Kluza, Philippe Marchetti

**Affiliations:** ^1^ Unit 837 Equipe 4 Inserm and Faculté de Médecine, Université de Lille II 1 Place Verdun 59045 Cedex, France; ^2^ EA 4483 Faculté des Sciences Pharmaceutiques et Biologiques de Lille 3, rue du Professeur Laguesse BP83 59006 Lille Cedex; ^3^ Laboratoire SPCMIB, UMR CNRS 5068 Université Paul Sabatier, 118 route de Narbonne, 31062 Toulouse Cedex 9, France; ^4^ INSERM U1065, Centre Méditerranéen de Médecine Moléculaire 151 route Saint-Antoine de Ginestière 06204 Nice cedex 3; ^5^ Roche SAS 30 cours de l'ile Seguin 92650 Boulogne Billancourt Cedex; ^6^ Centre de Bio-Pathologie, Plate-forme de Biothérapie, Banque de Tissus, CHRU Lille, France

**Keywords:** metabolism, oxidative phosphorylation, elesclomol, ROS

## Abstract

Vemurafenib/PLX4032, a selective inhibitor of mutant BRAFV600E, constitutes a paradigm shift in melanoma therapy. Unfortunately, acquired resistance, which unavoidably occurs, represents one major limitation to clinical responses. Recent studies have highlighted that vemurafenib activated oxidative metabolism in BRAFV600E melanomas expressing PGC1α. However, the oxidative state of melanoma resistant to BRAF inhibitors is unknown. We established representative *in vitro* and *in vivo* models of human melanoma resistant to vemurafenib including primary specimens derived from melanoma patients. Firstly, our study reveals that vemurafenib increased mitochondrial respiration and ROS production in BRAFV600E melanoma cell lines regardless the expression of PGC1α. Secondly, melanoma cells that have acquired resistance to vemurafenib displayed intrinsically high rates of mitochondrial respiration associated with elevated mitochondrial oxidative stress irrespective of the presence of vemurafenib. Thirdly, the elevated ROS level rendered vemurafenib-resistant melanoma cells prone to cell death induced by pro-oxidants including the clinical trial drug, elesclomol. Based on these observations, we propose that the mitochondrial oxidative signature of resistant melanoma constitutes a novel opportunity to overcome resistance to BRAF inhibition.

## INTRODUCTION

Activating mutations in BRAF, such as BRAFV600E can lead to aberrant MAPK signalling and proliferation in human tumors including melanoma, papillary thyroid carcinoma, and gastrointestinal stromal tumor [1-3]. Recently, targeting of mutants BRAF has emerged as a promising therapeutic strategy in these cancers. Results of phase II and III clinical trials with the first BRAF mutant selective inhibitor validated in clinic, vemurafenib/PLX4032, have revealed an impressive short-term disease stabilization in melanoma patients with BRAFV600E mutation [4,5]. Despise its promise, the major drawback of BRAF inhibition therapy, which has not yet been solved, is the apparition of resistance that inevitably occurs in patients even after an initial striking response [4]. Multiple molecular mechanisms of acquired resistance have been described culminating in the reactivation of the MAPK signaling pathway associated or not with the aberrant activation of the Akt pathway [6]. That includes the compensatory upregulation of receptor tyrosine kinases (such as PDGFRβ or IGFR1), activation of downstream kinases through oncogenic mutations of RAS or MEK, and upregulation of MAP3K8/COT or C-RAF kinases (for review [7,8]). Given the diversity of mechanisms, overcoming resistance to BRAF inhibitors remains challenging. Inhibition of mutant RAS has not yet resulted in effective therapeutic strategy [9]. MEK inhibitors have been unsuccessful both in preclinical models and in patients with resistance to BRAF inhibitors [10] suggesting that other compensatory pathways would be involved and, to date, no effective therapy that circumvents melanoma resistant to BRAF inhibitors is available. Thus, these observations highlight urgent need to find new therapeutic strategies to overcome resistance to BRAF inhibitors.

It is widely admitted that most cancer cells exhibit specific metabolic phenotypes that allow them to highly proliferate and survive to adverse environmental conditions [11]. Lessons from the last decade indicate that metabolic profile of cancer is much more heterogeneous than expected because metabolic pathways are intrinsically driven by oncogenic mutations, tumour suppressor gene inactivation and aberrant activation of proliferative pathways [12]. We and others have previously observed that metastatic melanomas are characterized by their strict dependence on glucose and glutamine for proliferation [13,14]. In approximately 90% of melanomas, this metabolic phenotype is associated with low mitochondrial bioenergetics activity [13,15,16]. However, the metabolic machinery of melanoma cells is not rigid and mitochondria are likely to have a key role in the metabolic flexibility of melanoma. In line with this, inhibition of the HIF/PDK signalling axis or overexpression of the key transcriptional cofactor in mitochondrial biogenesis, PGC1α, can restore mitochondrial oxidative metabolism in melanoma [13,15,17]. This latter is particularly relevant since PGC1α expression is transcriptionally controlled by the oncogenic melanocyte lineage-specification transcription factor, MITF, in a minor subset of melanomas [15].

It has been recently shown that MAPK activation slows down mitochondrial oxidative metabolism by repressing the MITF/PGC1α pathway [18]. Conversely, BRAF inhibitors stimulate mitochondrial oxidative phosphorylation thereby promoting ROS production in melanoma cells [15,18]. The oxidative metabolism can be considered as an adaptive mechanism that limits the efficacy of BRAF inhibitors [18]. In the current study, we examined mitochondrial metabolism and ROS production in several melanoma cell lines that exhibit acquired resistance to the BRAF inhibitor, vemurafenib. We have observed that BRAF inhibitor-resistant melanomas develop an addiction to mitochondrial oxidative metabolism characterized by high levels of basal mitochondrial respiration and ROS production. This metabolic phenotype, which is present irrespective of the expression of PGC1α, renders BRAF inhibitor–resistant melanoma cells highly vulnerable to several mitochondrial-targeted compounds including the mitochondrial pro-oxidative drug, elesclomol. These findings have particular implications for the development of new therapeutic strategies to eradicate melanomas that become resistant to BRAF inhibitors.

## RESULTS

### Mitochondrial metabolism and ROS production are induced by vemurafenib in BRAFV600E mutant melanoma cell lines irrespective of the PGC1α status

**Figure 1:**
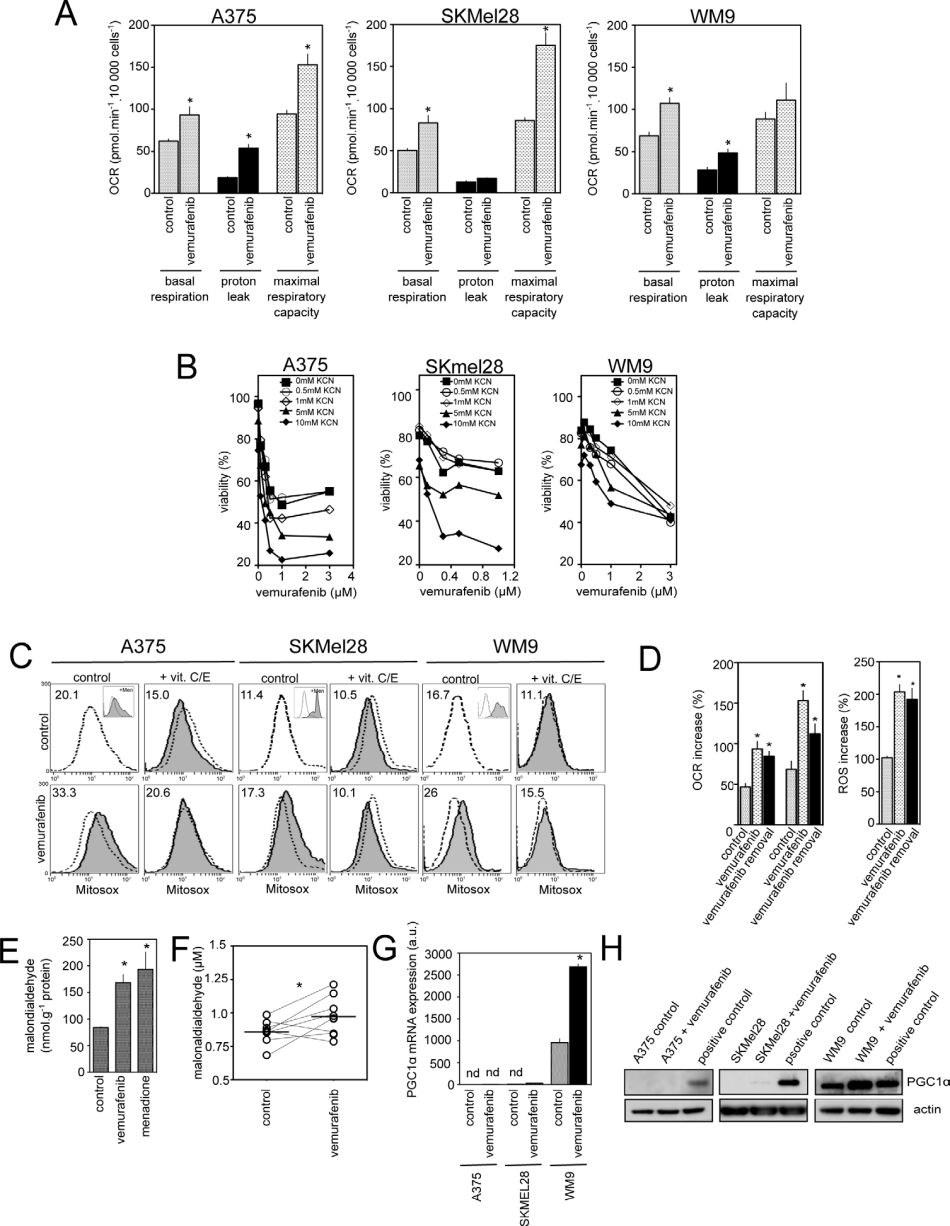
Effect of vemurafenib on mitochondrial oxidative metabolism in PGC1α positive and negative melanoma cell lines (A) Comparison of respiratory states (basal respiration, proton leak, maximum respiratory capacity) in melanoma cells (A375, SKMel28 and WM9) in the absence (control) or presence of vemurafenib (3μM for 24h) (see Material and Methods) *P<0.05 compared to control; (B) Viability of melanoma cells exposed to mitochondrial inhibitors KCN (0.5 to 2mM). Cell viability was estimated by PI after 72 h of treatment (mean+/−SD of three independent experiments); (C) Mitochondrial ROS production in melanoma cells exposed to vemurafenib. Representative flow cytometric profiles of melanoma cells exposed to 3μM vemurafenib in the presence or absence of 100 μM Vitamin C and E for 24 h. Cells were then stained with MitoSox before analysis. As positive control, cells were treated with 100μM menadione for 90 min (inset). Dashed line: fluorescence of control (untreated) cells. Numbers are the mean MitoSox fluorescence intensity values. Data represent typical results of one out of five independent experiments; (D) A375 cells were either kept untreated (control), treated with vemurafenib for 24h (vemurafenib), or exposed to vemurafenib for 24 h then washed and maintained for additional 24 h without vemurafenib (vemurafenib + withdrawal) before proceeding to determination of oxygen consumption (left) and ROS production (right). Data are means+/−SD of two experiments in duplicates. *P<0.05 compared to control (E) MDA levels were determined in A375 cells exposed to vemurafenib or kept untreated (control). As positive control of lipid peroxidation, cells were exposed to menadione as above. Data are means+/−SD of four independent experiments. *P<0.05 compared to control; (F) MDA levels were evaluated in blood plasma of 8 patients with BRAFV600E melanomas before vemurafenib and after 30 days of treatment. Horizontal lines are median values. *P<0.05 compared to control; (G) A375, SKMel28 or WM9 were exposed to 3μM vemurafenib for 24 h then total RNA were subjected to Q-RTPCR to quantify PGC1α mRNA abundance. Results are mean+/−SD of three independent experiments. Nd stands for not detectable. *P<0.05 compared to control; (H) Immunoblotting of PGC1α expression in melanoma cells treated with vemurafenib as in (G). Actin served as loading control.

**Figure 2:**
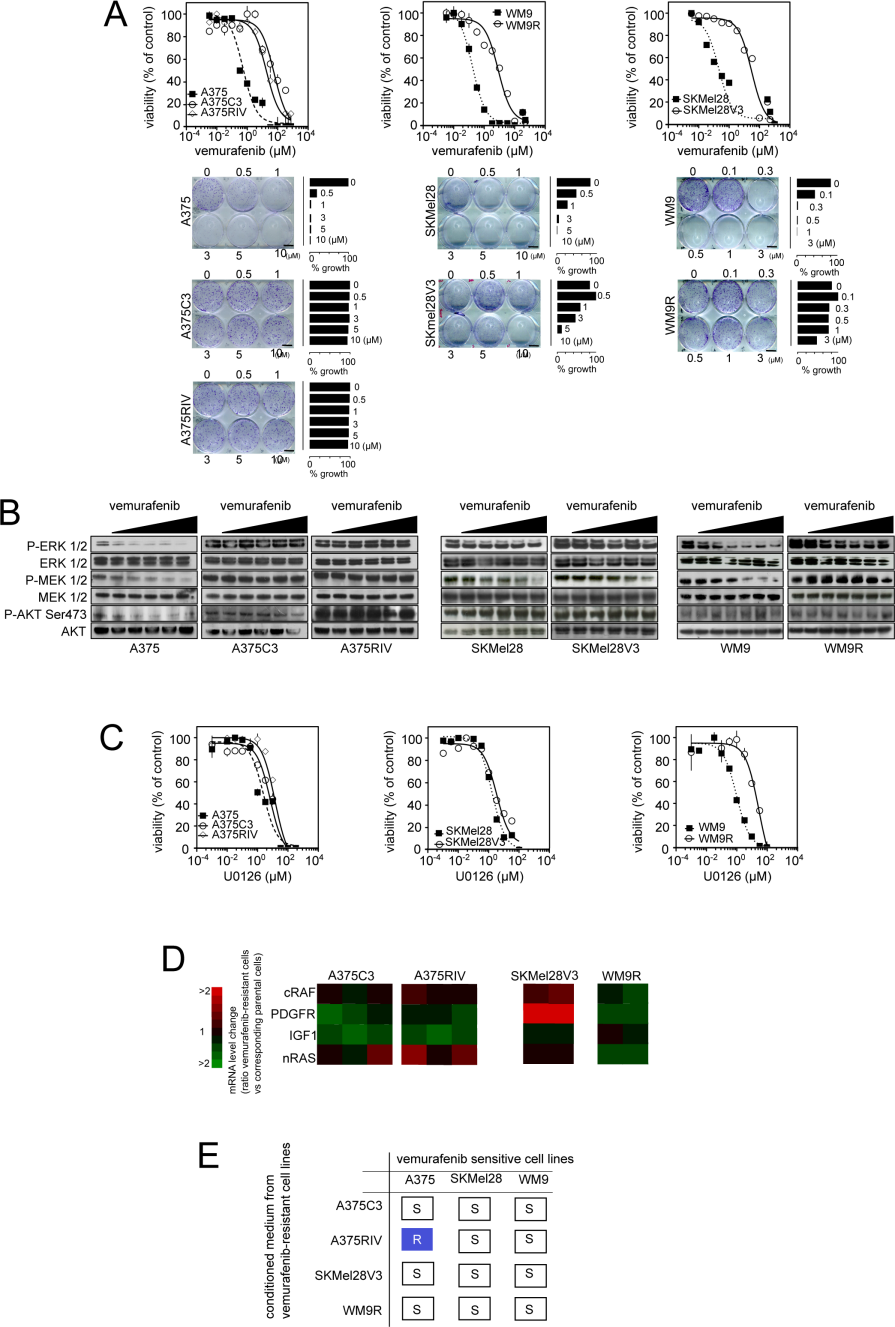
Generation of melanoma models of vemurafenib acquired resistance mediated through diverse mechanisms (A) *Upper panel*: Parental and vemurafenib-resistant cell lines were treated with increasing concentrations of vemurafenib (3 nmol/l to 750 μmol/l) for 3 days before the assessment of cell growth by MTS assay; *lower panel*: Colony-forming ability of A375, A375/C3 and A375/RIV treated with indicated doses of vemurafenib for 10 days. Photographs and relative quantification are representative of one experiment made in triplicates; (B) Effects of vemurafenib exposure (for 6 h, at the following doses: 100 nM, 300 nM, 500 nM, 1 μM, 3 μM and when indicated 5 μM) on the MAPK signaling cascade were evaluated by western blotting; (C) Parental and vemurafenib-resistant cell lines were treated with increasing concentrations of the MEK inhibitor, U0126, (1 nmol/l to 333 μmol/l) for 3 days before the assessment of cell growth by MTS assay; (D) Comparison of mRNA expression of N-Ras, C-Raf, IGF-1R, PDGFRβ between parental and vemurafenib-resistant melanomas; (E) Transmission of vemurafenib resistance from resistant to parental cells incubated for 4 h in conditioned medium from resistant sublines then treated for 72 h with 5 μmol/l vemurafenib before assessment of viability by flow cytometry. Summary of 3 independent experiments R : Resistance and S : sensitive.

Consistent with previous data [18], suppression of BRAFV600E signalling by vemurafenib exposure increased the oxygen consumption rate (OCR), an indicator of OXPHOS, in the BRAFV600E mutant human melanoma cell lines, A375, SKMel28 and WM9 (Fig. 1A). At concentrations inhibiting the MAPK pathway (Fig. 2B), vemurafenib exhibited both a higher basal OCR and a higher maximum respiratory capacity in comparison to untreated cells (Fig. 1A). The respiration inhibitor, KCN, enhanced vemurafenib-induced cell death in a dose-dependent manner indicating that OXPHOS is a limiting factor of the efficacy of vemurafenib (Fig. 1B and [18]). Since ROS are generated as by-products of the mitochondrial electron transport chain activity, we checked whether vemurafenib-increased respiration is associated with cellular ROS elevation. Mitochondrial superoxide detected with MitoSOX reagent was significantly increased in BRAFV600E mutant melanoma cell lines after vemurafenib exposure, an effect largely prevented by pretreatment with the antioxidants VitC and VitE (Fig. 1C). The increase in mitochondrial respiration and ROS generation persisted even 24h after vemurafenib removal (Fig. 1D). Moreover, the levels of malondialdehyde (MDA), a biomarker of ROS-dependent lipid peroxidation, were enhanced in A375 melanoma cells after exposition to vemurafenib (Fig. 1E) as well as in plasma after one-month treatment of patients with vemurafenib (Fig. 1F). Unlike WM9 cell line, the commercially available melanoma cell lines A375 and SKMel28 did not express significant level of PGC1α (Fig. 1G, 1H and [15,18]). Collectively, it appears that vemurafenib increases mitochondrial respiration and oxidative stress in BRAFV600E mutant melanoma cell lines including those that fail to express PGC1α.

### Vemurafenib-resistant melanoma cells exhibit high dependence on mitochondrial activity and constitutive oxidative stress

We next generated the acquired vemurafenib-resistant sub-lines (A375C3, SKMel28V3, WM9R) by prolonged exposure of the parental cell lines to vemurafenib. In an attempt to simulate the physiological apparition of resistance, we also constantly treated A375-xenografts in SCID mice with vemurafenib until the emergence of resistance then obtained “in vivo” the resistant sub-line, A375RIV (Supplemetary Fig. 1A). This protocol phenocopies the situation observed in patients. Unlike the parental sensitive cells, we confirmed the resistant status of A375C3, A375RIV, SKMel28V3, WM9R sub-lines in cell survival (Fig. 2A, upper panel) and longer-term clonogenic assays (Fig. 2A, lower panel). No elevated levels of BRAF or alternatively splices isoforms (61 kDa, [19]) were found in the resistant sub-lines (Suplementary Fig. 1B). Genomic analyses did not revealed classical secondary mutations such as NRAS^Q61K^, KRAS^K117N^, MEK^C121S^ in the resistant sub-lines (Supplementary Table 1). To further explore the mechanisms of resistance, we examined the activation status of BRAF downstream targets, MEK and ERK, as well as the RTK-dependent stimulation of Akt in vemurafenib-sensitive and derived-resistant sub-lines (Fig. 2B). Vemurafenib caused dose-dependent decreases in p-MEK1/2 and p-ERK1/2 in all sensitive cell lines whereas all 4 resistant sub-lines maintained elevated levels of p-MEK1/2 and p-ERK1/2 upon vemurafenib exposure. However, the levels of p-MEK1/2 and p-ERK1/2 as well as those of p-Akt differed among the resistant sub-lines suggesting distinct mechanisms of resistance (Fig. 2B). Besides, only WM9R cells remained resistant to the MEK1/2 inhibitor, U0126, indicating that they activated new survival signalling pathways in the presence of vemurafenib (Fig. 2C). In addition, PDGFRβ mRNA was found upregulated in the SKMel28V3 resistant sub-line insinuating that the activation of a PDGFRβ-dependent pathway is involved in SKMel28V3 resistance to vemurafenib (Fig. 2D). Since activation of multiple RTKs by various ligands can be responsible for acquired resistance to kinase inhibitors [20], we tested the effect of conditioned media from vemurafenib-resistant sub-lines on sensitivity to vemurafenib in parental cells. Of note, only the A375RIV conditioned medium transferred vemurafenib resistance to the parental A375 cells suggesting that resistance of A375RIV was sustained by autocrine/paracrine survival mechanisms (Fig. 2E). Thus, although the precise mechanisms remain to be determined, we have generated a panel of human melanoma cell lines with diverse mechanisms of acquired resistance to vemurafenib.

**Figure 3:**
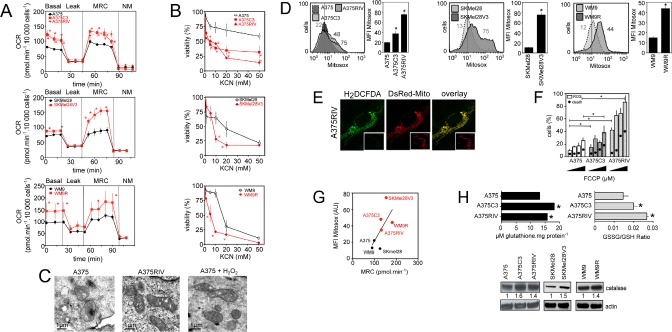
Mitochondrial oxidative stress in vemurafenib resistant cells (A) Oxygen consumption rate (OCR pmol/min) in vemurafenib resistant melanoma cell lines in comparison to parental cells. The different states of mitochondrial respiration are indicated: basal respiration (Basal), proton leak (respiration after oligomycin exposure), maximal respiratory capacity (respiration after FCCP, MRC), non-mitochondrial respiration (after rotenone and antimycin A) (NM). *P<0.05 compared to control; (B) Effect of inhibition of respiration on viability of parental and vemurafenib-resistant cells; Cells were exposed to indicated doses of KCN for 24 h then viability was assessed by flow cytometry after PI staining. *P<0.05 compared to control (C) Morphology of mitochondria in A375 and A375RIV cells by transmission electron microscopy. As a control, A375 cells were treated with 500 μmol/l H_2_O_2_ for 1h. Scale bar: 1 μm; (D) Mitochondrial ROS production in parental and vemurafenib-resistant cell lines. Representative flow cytometric profiles (left) and histogram (right, mena +/−SD) of five independent experiments. Cells were then stained with MitoSox before analysis. *P<0.05 compared to control; (E) H2DCFDA staining (*green*) co-localizes with DsRed-labelled mitochondria (*red*) in A375RIV cells. Typical fluorescence images of one experiment. (Inset) DsRed-labelled mitochondria without H2DCFDA staining; (F) Effects of FCCP (2, 5, 10 μM) on ROS production and cell death on A375 and A375C3, A375 RIV. Cells were treated for 6h before ROS determination by flow cytometry as described above and for 48 h before assessment of cell death by PI staining. (Results are means +/−SD from 3 independent experiments). *P<0.05 compared to control; (G) Correlation between mitochondrial activity (MRC) and ROS production (MFI MitoSox values) in several human melanoma cell lines; (H) Determination of the antioxidant status in A375 and A375 C3, A375 RIV cells. (*upper panel*) determination of total glutathione and GSSG/GSH ratio as described in Materials and methods. Data are means +/− SD of three independent experiments. *P<0.05 compared to control; (*lower panel*) Expression of catalase analysed by immunoblotting. Actin served as loading control. Representative images of three independent experiments. Mean values obtained from densitometric measures and normalized to the actin values are represented.

In these models of vemurafenib resistance, we next investigated the dependence of cells on mitochondrial oxidative metabolism (Fig. 3). First, both routine respiration and maximum respiratory capacity (respiration stimulated with FCCP) were significantly enhanced in the four vemurafenib-resistant sub-lines compared to their parental counterparts (Fig. 3A). Consistent with their high mitochondrial metabolism, vemurafenib-resistant cells were more sensitive than parental cells to the lethal effect of the complex IV inhibitor, KCN (Fig. 3B) suggesting that melanoma resistant to BRAF inhibitors largely depend on mitochondrial metabolism for survival. We also noticed that vemurafenib-resistant cells presented a low content in several TCA intermediates and in lactate level, an observation compatible with high mitochondrial activity (Table 1). As previously observed [18], vemurafenib increased the mitochondrial content of melanoma cells sensitive to BRAF inhibitors (Supplementary Fig. 2B). Conversely, the mitochondrial biogenesis response was not affected in vemurafenib-resistant cells (Supplementary Fig. 2A and 2B). Although vemurafenib-resistant cells did not have more mitochondria, qualitative examination of mitochondria by electron microscopy revealed morphological changes including more cristae with wide intracristal spaces in vemurafenib resistant cells compared to sensitive cells (Fig. 3C). Consistent with the high mitochondrial activity observed in vemurafenib-resistant cells, these cells generated more mitochondrial ROS (assessed by flow cytometric analysis of MitoSox fluorescence) than their sensitive counterparts (Fig. 3D). As revealed by the overlap of ROS-dependent H2DCFDA fluorescence and DsRedMito fluorescence, we confirmed that mitochondria are an important source of ROS in vemurafenib-resistant cells (Fig. 3E). Similar ROS increasing pattern was observed in vemurafenib-resistant cells after transfection with Hypermito, a genetically encoded probe for specific detection of mitochondrial hydrogen peroxide [21] (Supplementary Fig 2D). Addition of the uncoupler FCCP maximized mitochondrial respiration (Fig 1A and 3A) and increased mitochondrial ROS (Fig. 3F). The pro-oxidative effect of FCCP was more pronounced in vemurafenib-resistant cells than in parental cells, in agreement with its higher effect on the respiration of vemurafenib-resistant cells (Fig. 3A). Enhanced respiration correlated well with the increase in ROS generation in melanoma cell lines (Spearman R=0.85, p=0.02) confirming the mitochondrial origin of ROS in these cells (Fig. 3G). Consistent with oxidative stress, the ratio of oxidized to reduced glutathione (GSSG/GSH) was elevated in vemurafenib-resistant cells compared to parental cells (Fig. 3H). This was associated with a slight increase in the amount of total glutathione (Fig. 3H) and of the level of the anti-oxidant enzyme, catalase, in vemurafenib-resistant cells. We conclude, therefore, that mitochondrial oxidative stress is a cellular characteristic of melanoma cells that have acquired resistance to vemurafenib.

**Table 1:** Metabolites quantification by H-NMR in vemurafenib-resistant A375C3 and A375 cells

(nmol/mg)	Lactate	Citrate	Pyruvate	Fumarate	Malate	Succinate
A375	677.45+/− 57.96	17.39+/−5.35	1.02+/−0.08	2.48+/−0.23	36+/−4.25	15.08+/−1.51
A375C3	*557.45+/−13.97	11.83+/−2.38	*0.89+/−0.07	*1.32+/−0.12	*16.96+/−2.55	*11.59+/−1.78

N=5. Results are means +/− SD.

* P< 0.05 versus A375

### Vemurafenib-resistant melanoma cells are prone to cell death induced by the pro-oxidant elesclomol

**Figure 4:**
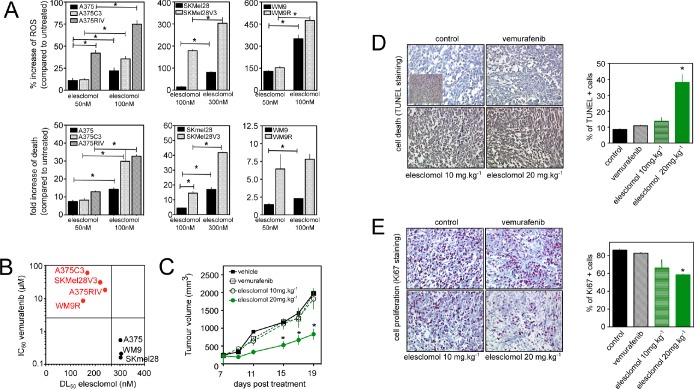
Effects of the pro-oxidant elesclomol on vemurafenib-resistant melanoma cells (A) ROS generation (determined by flow cytometry, upper panel) and cell death (determined by PI staining lower panel) induced by elesclomol at the indicated doses for 6h in A375, A375C3 and A375RIV cell lines and for 3h in other melanoma cell lines. Data are means +/− SD of two independent experiments made in duplicates. *P<0.05 compared to control; (B) Scatterplot melanoma cell lines of the sensitivity toward vemurafenib (determination of IC50 values after 72h of treatment) and elesclomol (determinion of DL 50 values after 6h of treatement); (C) *In vivo* efficacy of elesclomol in tumor-bearing mice. A375C3 cells were injected into the right flank of SCID mice. Mice were treated either with vemurafenib 75mg/kg seven days a week by oral gavage or with elesclomol 10mg/kg or 20mg/kg *i.v*. Tumour volume was measured at the indicated times. Data represent means +/−SD from 6 to 10 mice per group. *P<0.05 compared to control. Histological sections from tumor-bearing mice were labelled with an anti-Ki67 antibody (D) to detect cell proliferation and by TUNEL assay (E) to assess cell death (mean+/−SD, n=3, *P<0.05 compared to control).

**Figure 5:**
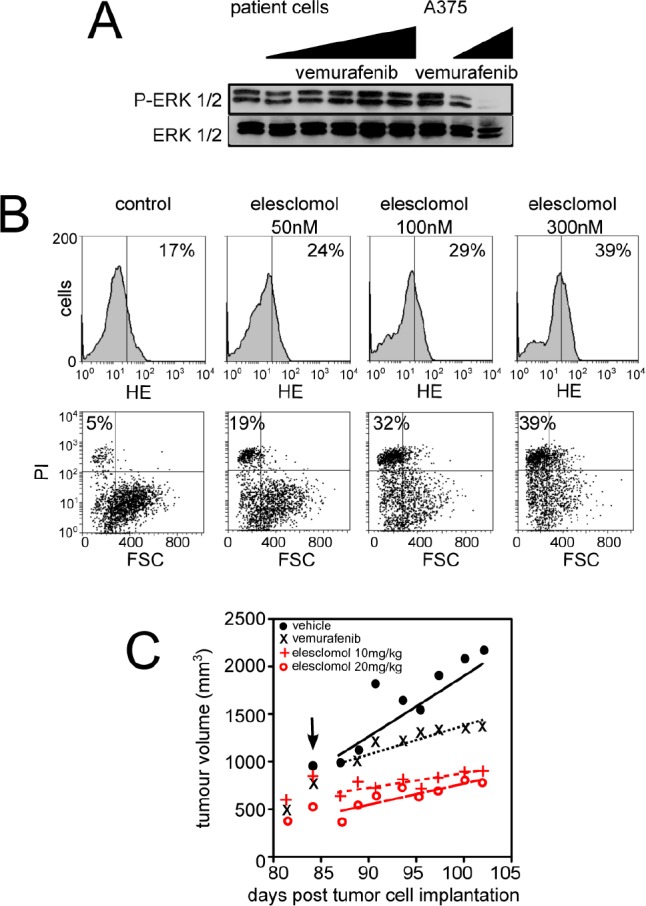
Effects of elesclomol on primary melanoma cells from patient resistant to vemurafenib (A) Western blot analysis of the effects of vemurafenib exposure (for 6h at increasing doses) on ERK phosphorylation in melanoma cells derived from one patient with acquired resistance to vemurafenib. A375 cells were also used as control; (B) Effect of elesclomol on ROS production (upper panel) and cell death (lower panel) in melanoma cells derived from one patient with acquired resistance to vemurafenib; (C) Graph representating growth of tumor xenograft from the same patient than in B. Tumor xenografts were treated (black arrow) following the above protocol.

The observation that resistance to vemurafenib inevitably occurs in melanoma cells points out the need to develop new strategies to kill vemurafenib-resistant cells by other mechanisms. Since FCCP induced cell death preferentially in vemurafenib-resistant cells (Fig. 3G), we hypothesized that the constant increase in ROS production observed in vemurafenib-resistant cells would render these cells more sensitive to further oxidative stress by exogenous agent. We tested this possibility using elesclomol, a pro-oxidative drug that displays clinical anti-melanoma activity [22]. Elesclomol has been shown to pick up electrons from the mitochondrial ETC and the subsequent electron leakage causes oxidative stress [23]. Consistent with our previous data [13], elesclomol induced a dose-dependent increase in intracellular ROS generation and melanoma cell death (Fig. 4A). Interestingly, cell death induced by elesclomol was remarkably higher in vemurafenib-resistant cell lines than in parental cells (Fig. 4A and 4B), consistent with the high level of mitochondrial oxidative stress observed in resistant cells (Fig. 3). High oxidative toxicity was also observed in vemurafenib-resistant cells incubated with other pro-oxidative drugs such as the mitochondrial ROS inducers, menadione, and phenylethyl isothiocyanate (PEITC) (supplementary Fig. 3A and 3B). We then set out to determine the antimelanoma effects of elesclomol *in vivo* in SCID mice xenografted with the vemurafenib-resistant human cell line, A375C3. Whereas A375C3 tumors continued to grow despite treatment with vemurafenib, animals treated with elesclomol had significantly smaller A375C3 tumors (Fig. 4C). The *in vivo* effect of elesclomol on tumor growth was associated with the occurrence of apoptosis (Fig. 4D) and the decrease in cell proliferation (Fig. 4E). Besides, substantial increase of ROS and cell death was also observed *in vitro* after elesclomol exposure in cells isolated from a patient with metastatic BRAFV600E-bearing melanoma, who escaped to treatment with vemurafenib (Fig. 5A and 5B). The ability of elesclomol to reduced melanoma growth was finally confirmed *in vivo* by engrafting SCID mice with vemurafenib-resistant tumor fragments obtained from the same patient (Fig. 5C). Overall, melanomas with acquired resistance to vemurafenib remain sensitive to the pro-oxidant, elesclomol suggesting that mitochondrial pro-oxidants may have a potential for treatment of vemurafenib-resistant melanoma in the clinic.

## DISCUSSION

Like many types of cancer, the vast majority of melanomas adopts a Warburg phenotype that renders them predominantly dependent on aerobic glycolysis for survival and proliferation [13-15]. However, it is now evident that mitochondria in human melanoma cells do not remain inactive and exhibit specific functional characteristics. Melanoma cells maintain entirely functional mitochondria that can metabolize carbons derived from glutamine and glucose for anabolic purposes [14]. This is further supported by the observation that mitochondrial oxidative metabolism can be unlocked by inhibition of the HIF-1/PDK signalling pathway [13]. Very recently, two reports have revealed key mechanisms of melanoma oxidative metabolism [15,18]. The metabolic phenotype of melanoma is heterogeneous and is largely dependent on the expression of the positive regulator of mitochondrial biogenesis, PGC1α, found in approximately 10% of melanoma [15]. PGC1α positive cells display high rates of oxidative phosphorylation and appear to be addicted to oxidative metabolism for survival [15]. Mechanistically, the expression of PGC1α is directly under control of the lineage specific transcription factor, MITF [15,18] and the activating BRAFV600E mutation suppress MITF expression, which, in turn, reduces PGC1α and subsequently curtails mitochondrial metabolism [18]. As predicted by these relationships, inhibition of B-RAF with specific inhibitors such as vemurafenib increases the expression of MITF and PGC1α [18]. Our results complement these data [18] demonstrating that vemurafenib increases mitochondrial respiration and ROS generation in melanoma cells. Our results also indicate that BRAF inhibitors can increase mitochondrial metabolism through a PGC1α independent mechanism. Multiple factors can influence mitochondrial metabolism. Lessons from the PGC1α null mice indicate that PGC1α do not appear to be the sole determinant of mitochondrial biogenesis [24] and other proteins of the PGC1 family including PGC1β and the PGC1-related coactivator (PRC), are regulated coactivators which may boost respiration to meet energy needs associated with cell growth [25]. Irrespective of the mechanisms, we have shown that pre-incubation with inhibitors of mitochondrial function renders melanoma cells more sensitive to BRAF inhibitors. Haq and colleagues [18] have also recognised mitochondrial activity as a significant hurdle to the cytotoxic effect of vemurafenib. In line with this, the Bcr-Abl specific inhibitor, imatinib stimulates mitochondrial activities contributing to limit its anti-leukemia effects [26]. Since mitochondrial oxidative metabolism triggered by vemurafenib constitutes a brake on the cytotoxicity induced by BRAF inhibitors, one can hypothesize that cells that can survive in the presence of vemurafenib are those characterized by high mitochondrial activity.

Herein, we have identified mitochondrial oxidative metabolism as a metabolic signature of resistance to BRAF inhibitors. We employed four independent models of resistance for this investigation in an attempt to better mimic diversity in mechanisms of acquired resistance in the clinic. We have observed that vemurafenib-resistant cells, which sustain high levels of MAPK signalling, displayed a significant increase in respiration rates as well as high levels of mitochondrial superoxide anion compared to sensitive cells. This is consistent with the observation that the enforced expression of oncogenic BRAFV600E in fibroblasts is sufficient to promote mitochondrial oxidative metabolism characterized by high level of pyruvate oxidation, respiration and rise in oxidative stress [17]. Interestingly, the mitochondrial oxidative phenotype has been involved in the resistance to classical chemotherapy and BRAF inhibitors in the slow-cycling subpopulation of melanoma characterized by high expression of the lysine-specific demethylase 5B (KDM5B *a.k.a*. JARID1B) [27]. As previously suggested [28], response to oncogene inhibition depends not only on driven oncogenes but also on adaptative conditions selected during melanomagenesis.

Cancer metabolism is now considered as an emerging source of new targets for cancer therapy [29]. These results identify mitochondria as potential targets for the treatment of melanoma resistant to BRAF inhibitors. Vemurafenib-induced oxidative metabolism renders melanoma cells highly dependent on antioxidant enzymes to cope with oxidative stress. One can then hypothesize that vemurafenib-resistant cells, which survive in the presence of vemurafenib, possess sufficient adaptive antioxidant mechanisms to tolerate the chronic excess of ROS. Thus, it is not surprising that we observed an increase in the level of glutathione and catalase in vemurafenib-resistant cells. In this situation, a further oxidative stress (*e.g.* induced by pro-oxidative drugs) could exhaust the antioxidant defence and push cells beyond the oxidative level where cell death can occur [30]. This may explain why vemurafenib-resistant cells with increased endogenous ROS are more sensitive to cell death induced by mitochondrial pro-oxidative agents. Since cell lines resistant to vemurafenib displayed an important activity in the respiratory chain, we have exposed them to the pro-oxidative drug, elesclomol. Elesclomol combined with copper targets the mitochondrial electron chain and induces a respiratory-dependent ROS production [23]. Elesclomol was evaluated in a Phase III clinical trial for the treatment of metastatic melanoma with encouraging results [22] and is currently being evaluated in a Phase I trial in the treatment of AML (clinicaltrials.gov).

Overcoming resistance to BRAF inhibition is currently a critical area of investigation. Results obtained in recent years suggest that resistance to vemurafenib can occur by multiple distinct mechanisms that are totally unpredictable. In our present study, we suggest a global strategy consisting to exploit a general hallmark of melanoma cells that have acquired resistance to vemurafenib regardless the mutation profile. In addition to increasing pro-oxidative stress, HSP90 inhibition or ER stress inducers have been also shown to be valuable therapeutic targets in BRAF mutant melanoma [31,32] enabling to overcome acquired resistance to vemurafenib [32,33].

In conclusion, we propose a new paradigm in therapeutic strategy aimed at increasing mitochondrial oxidative stress to eradicate melanoma resistant to BRAF inhibitors.

## MATERIALS AND METHODS

### Reagents

Reagents were purchased from Sigma-Aldrich (StLouis, MO, USA) unless otherwise stated. Vemurafenib (PLX4032) was from Roche, elesclomol from Synta Pharmaceuticals Corp. and U0126 from SelleckChem (Euromedex, Souffelweyersheim, France).

### Clinical specimen

This study has received an ethical approval of the local Person's Protection Committee. All patients were recruited from the Department of Dermatology, Lille CHRU, France) and gave informed consent. Skin melanoma metastasis samples from one patient with acquired resistance to vemurafenib were obtained after informed consent. Molecular analysis of the tumor confirmed the presence of a BRAFV600E mutation. Of these samples, four were used for *in vivo* experiments and the remaining samples were used for *in vitro* experiments as described below. Besides, blood plasma samples were obtained from 8 patients with BRAFV600E mutant melanoma the day before and 30 days after vemurafenib at a dose of 960 mg twice daily.

### Cell culture and derivation of vemurafenib-resistant cell lines

A375 and SKMel28 human melanoma cell lines were purchased from the American Type Culture Collection and WM9 human melanoma cell line was obtained by a kind gift from a Dr. M. Herlyn (The Wistar Institute, Philadelphia). All cell lines have been found to harbour BRAFV600E mutation. To generate cell line with in vitro acquired resistance, BRAFV600E mutant cell lines (A375, SKMel28, WM9) sensitive to vemurafenib, were treated with different concentrations (approximately 3×IC50, 10×IC50 of the sensitive cell line) of vemurafenib for 2-3 months until a subline grew progressively as described [10,34]. Vemurafenib-resistant cells were cloned in 3μM vemurafenib, a concentration at which parental cells were not viable. Vemurafenib-resistant cells obtained *in vitro* were designated A375C3, SKMel28V3 and WM9R. Trypan blue exclusion assay was regularly performed to check resistance status. For the obtention of the A375RIV vemurafenib-resistant cell line, we used human melanoma xenograft models in which drug resistance is selected by continuous vemurafenib administration in immunocompromised mice (Supplementary Fig. S1). Briefly, A375 orthotopic tumors were grown to 300 mm3 before treatment with vemurafenib (75mg/kg/day by oral gavage). Tumor growth was inhibited for 45 days of treatment, at which time one tumor rapidly progressed. The occurrence of resistance is in line with clinical data in humans. Once this tumor reached a volume of 1500 mm3, the mouse was euthanized, and tumor tissue was removed and primary cell culture was established. Tumor tissue was minced in a sterile glass Petri dish then incubated at 37°c for 150 minutes with an enzyme cocktail of serum-free DMEM (Invitrogen, San Diego, CA) containing 8 mg/ml collagenase-1 and 5 mg/ml dispase. This mixture was incubated for an additional 30 minutes with 1ml of trypsin 0.05%. The enzymatic reaction was stopped by addition of DMEM supplemented with 10% FCS and penicillin/streptomycin (Invitrogen). Cells were grown to allow elimination of contaminating fibroblasts before further study. Cell lines were maintained in RPMI (except for SKMEL28, SKMEL28V3 in DMEM) with 10% FCS and were periodically tested for mycoplasma contamination.

### Mutational analysis of NRAS, BRAF, and MEK by direct PCR product sequencing

Mutational analysis of MAP2K1/C121S, NRAS/Q61K and KRAS/K117N was performed in all of vemurafenib-resistant cell lines using direct PCR product sequencing on the mutated hotspots of these genes. Purification, adaptators ligation, barcoding, template preparation (emPCR) were done on the One-Touch systen (Lifetechnologies) and sequencing reactions on the Ion Torrent PGM (Lifetechnologies) following manufacturer's recommandations.

### Immunoblotting

Cell lysates were prepared as described previously [13] then 20 μg proteins were separated on a 4-12% SDS-PAGE then transferred to nitrocellulose membrane. After blocking for 1 h in 10% BSA in TBS Tween buffer, membranes were probed with the following antibodies specific for Akt (1:1,000, #9272, Cell Signaling Technology Inc., Denvers, MA), phospho-Akt at Ser473 (1:1,000, 193H12, Cell Signaling Technology Inc.), p44/42 MAPK (Erk1/2) (1:1,000, #9102, Cell Signaling Technology Inc.), phospho-p44/42 MAPK (Erk1/2) at Thr202/Tyr204 (1:1,000, #9101, Cell Signaling Technology Inc.), MEK1/2 (1:1,000, #9122, Cell Signaling Technology Inc.), phosphor-MEK1/2 (1:1,000, #9121, Cell Signaling Technology Inc.). Horseradish peroxidase-conjugated secondary antibodies from Rockland Immunochemicals Inc. (Gilbertsville, PA) were used at 1:2,000 for 1h then detection was carried out by enhanced chemoluminescence. For detection of OXPHOS complexes, monoclonal antibodies from MitoSciences were used as described [35].

### Microscopic imaging

Indicated cell lines were transiently transfected with a plasmid encoding mitochondrially-targeted red fluorescent protein (pDsRed2-Mito, Clontech Laboratories Inc., 0.5μg/200,000 cells) using Lipofectamine PLUS (Invitrogen) according to the manufacturer's protocol. pDsRed2-Mito-transfected cells were seeded on 24mm glass coverslip for 24h before microscopic analysis (Leica DMR, Heidelberg, Germany). pDsRed2-Mito was excited at 594nm under x630 magnification before acquisition.

### Clonogenic assay and proliferation

Cells (500/well) were seeded into 6-well plates and treated with indicated doses of vemurafenib in different culture medium. After 10 days of culture, colonies were stained with crystal violet, digital images were taken, then colonies were de-stained in acetic acid (30%) before densitometric quantification with the SAFAS UVMc2 spectrophotometer (Safas Monaco).

### Cytofluorometric analysis

Evaluation of cell viability was performed following with propidium iodide staining[13,35]. Detection of ROS was assessed with several oxidation sensitive fluorescent probes such as hydroethidine (HE), mitochondria-targeted HE (Mito-SOX) and CM-H2DCFDA following classical protocols [13,35]. Alternatively, cells were transiently transfected with 1 μg of plasmid DNA encoding redox-sensitive green fluorescent proteins targeted to mitochondria (Hyper-mito, Evrogen) as described [21]. Fluorescence was analyzed on a FACS Canto II cytofluorometer (Beckton Dickinson).

### Glutathione status

Glutathione status (*i.e.* glutathione disulfide, GSSG/reduced glutathione, GSH) was determined in cell pellets by using high-performance liquid chromatography with fluorescence detection, as published [36].

### Determination of malondialdehyde (MDA)

MDA concentrations was determined in cell pellets and plasma samples by using high-performance liquid chromatography with fluorescence detection, as published [37].

### PCR analysis

Quantitative detection of mRNA was performed by real-time PCR using the Lightcycler 480 detector (Roche Applied Science, Manheim Germany) and comparison was done with the Pfafll method as described [35]. The transcript levels in triplicates were normalized to those of α4 tubulin. The sequences of primers are: PPARGC1A (PGC1α) sense 5'-CTGCTAGCAAGTTTGCCTCA-3' and antisense 5'-AGTGGTGCAGTGACCAATCA-3' and α4 tubulin 5'-GACAGCTCTTCCACCCAGAG-3' and antisense 5'-TGAAGTCCTGTGCACTGGTC-3'.

### Assessment of oxygen consumption

Respiratory capacity of melanoma cells were performed with the Seahorse XF24 Extracellular Flux Analyser (Seahorse Bioscience, Billerica, MA, USA) on attached cells as described [16]. Briefly, 2 × 10^4^ melanoma cells/well were seeded in XF24 V7 microplates for 24h before vemurafenib exposure. Before analysis, cells were resuspended in Seahorse assay buffer and the following drugs were added: 1 μM oligomycin, 0.25-0.5 μM FCCP, 1 μM rotenone and 1 μM antimycin A.

### In vivo study

All procedures with animals were performed according to institutional guidelines for use of laboratory animals (agreement provided by the Animal Care Ethical Committee). Immunodeficient female SCID mice, 6 to 8 wk old, under isoflurane anesthesia were injected with 2×10^6^ A375C3 cells, mixed (1 :1 volume) with BD Matrigel Basement Membrane Matrix. Tumor volume was calculated with a caliper by the standard formula L × l^2^ / 2. When tumors reached approximately 400 mm3, the mice were divided into four groups: Control group n=4: mice were treated with saline with the same schedule as the treated animals; Elesclomol 10 mg/kg group n=6 (elesclomol 10 mg/kg, i.v. injection for 5days/week); Elesclomol 20 mg/kg group n=6 (elesclomol 20 mg/kg, i.v. injection for 5days/week); vemurafenib group n=4 (vemurafenib administrated by oral gavage, 75 mg/kg/j). For patient-derived tumor implanted in mice, fresh tumor samples were minced into small pieces, mixed (1 :1 volume) with Matrigel then injected into the flank of SCID mice as described above.

### Histology

For *in situ* determination of cell proliferation or apoptosis, Ki-67 and TUNEL (In situ Cell Death Detection kit, Roche) staining were performed on histological sections as described [13].

### Statistical analysis

Statistics were performed with GraphPad Prism® version 5.00 (GraphPad Software, San Diego, CA, USA). Data are presented as the mean±SD. The student's t-test was used to compare data sets and Paired t-test to compare MDA before and after treatment. Statistical significance was set at P<0.05.

## Supplementary Figures and Tables





## References

[1] Dong J, Phelps RG, Qiao R, Yao S, Benard O, Ronai Z, Aaronson SA (2003). BRAF oncogenic mutations correlate with progression rather than initiation of human melanoma. Cancer Res.

[2] Falchook GS, Trent JC, Heinrich MC, Beadling C, Patterson J, Bastida CC, Blackman SC, Kurzrock R (2013). BRAF mutant gastrointestinal stromal tumor: first report of regression with BRAF inhibitor dabrafenib (GSK2118436) and whole exomic sequencing for analysis of acquired resistance. Oncotarget.

[3] Nucera C, Lawler J, Hodin R, Parangi S (2010). The BRAFV600E mutation: what is it really orchestrating in thyroid cancer?. Oncotarget.

[4] Chapman PB, Hauschild A, Robert C, Haanen JB, Ascierto P, Larkin J, Dummer R, Garbe C, Testori A, Maio M, Hogg D, Lorigan P, Lebbe C, Jouary T, Schadendorf D, Ribas A (2011). Improved survival with vemurafenib in melanoma with BRAF V600E mutation. N. Engl. J. Med.

[5] Sosman JA, Kim KB, Schuchter L, Gonzalez R, Pavlick AC, Weber JS, McArthur GA, Hutson TE, Moschos SJ, Flaherty KT, Hersey P, Kefford R, Lawrence D, Puzanov I, Lewis KD, Amaravadi RK (2012). Survival in BRAF V600-mutant advanced melanoma treated with vemurafenib. N. Engl. J. Med.

[6] McCubrey JA, Steelman LS, Chappell WH, Abrams SL, Franklin RA, Montalto G, Cervello M, Libra M, Candido S, Malaponte G, Mazzarino MC, Fagone P, Nicoletti F, Bäsecke J, Mijatovic S, Maksimovic-Ivanic D (2012). Ras/Raf/MEK/ERK and PI3K/PTEN/Akt/mTOR Cascade Inhibitors: How Mutations Can Result in Therapy Resistance and How to Overcome Resistance. Oncotarget.

[7] Alcalá AM, Flaherty KT (2012). BRAF Inhibitors for the Treatment of Metastatic Melanoma: Clinical Trials and Mechanisms of Resistance. Clinical Cancer Research.

[8] Corcoran RB, Settleman J, Engelman JA (2011). Potential therapeutic strategies to overcome acquired resistance to BRAF or MEK inhibitors in BRAF mutant cancers. Oncotarget.

[9] Posch C, Ortiz-Urda S (2013). NRAS mutant melanoma--undrugable?. Oncotarget.

[10] Nazarian R, Shi H, Wang Q, Kong X, Koya RC, Lee H, Chen Z, Lee M-K, Attar N, Sazegar H, Chodon T, Nelson SF, Mcarthur G, Sosman JA, Ribas A, Lo RS (2010). Melanomas acquire resistance to B-RAF(V600E) inhibition by RTK or N-RAS upregulation. Nature.

[11] Ward PS, Thompson CB (2012). Metabolic Reprogramming: A Cancer Hallmark Even Warburg Did Not Anticipate. Cancer Cell.

[12] Fritz V, Fajas L (2010). Metabolism and proliferation share common regulatory pathways in cancer cells. Oncogene.

[13] Kluza J, Corazao Rozas P, Touil Y, Jendoubi M, Maire C, Guerreschi P, Jonneaux A, Ballot C, Balayssac S, Valable S, Corroyer-Dulmont A, Bernaudin M, Malet-Martino M, de Lassalle EM, Maboudou P, Formstecher P (2012). Inactivation of the HIF-1α/PDK3 signaling axis drives melanoma toward mitochondrial oxidative metabolism and potentiates the therapeutic activity of pro-oxidants. Cancer Res.

[14] Scott DA, Richardson AD, Filipp FV, Knutzen CA, Chiang GG, Ronai ZA, Osterman AL, Smith JW (2011). Comparative Metabolic Flux Profiling of Melanoma Cell Lines: BEYOND THE WARBURG EFFECT. J Biol Chem.

[15] Vazquez F, Lim J-H, Chim H, Bhalla K, Girnun G, Pierce K, Clish CB, Granter SR, Widlund HR, Spiegelman BM, Puigserver P (2013). PGC1α expression defines a subset of human melanoma tumors with increased mitochondrial capacity and resistance to oxidative stress. Cancer Cell.

[16] Hall A, Meyle KD, Lange MK, Klima M, Sanderhoff M, Dahl C, Abildgaard C, Thorup K, Moghimi SM, Jensen PB, Bartek J, Guldberg P, Christensen C (2013). Dysfunctional oxidative phosphorylation makes malignant melanoma cells addicted to glycolysis driven by the V600EBRAF oncogene. Oncotarget.

[17] Kaplon J, Zheng L, Meissl K, Chaneton B, Selivanov VA, Mackay G, van der Burg SH, Verdegaal EME, Cascante M, Shlomi T, Gottlieb E, Peeper DS (2013). A key role for mitochondrial gatekeeper pyruvate dehydrogenase in oncogene-induced senescence. Nature.

[18] Haq R, Shoag J, Andreu-Perez P, Yokoyama S, Edelman H, Rowe GC, Frederick DT, Hurley AD, Nellore A, Kung AL, Wargo JA, Song JS, Fisher DE, Arany Z, Widlund HR (2013). Oncogenic BRAF regulates oxidative metabolism via PGC1α and MITF. Cancer Cell.

[19] Poulikakos PI, Persaud Y, Janakiraman M, Kong X, Ng C, Moriceau G, Shi H, Atefi M, Titz B, Gabay MT, Salton M, Dahlman KB, Tadi M, Wargo JA, Flaherty KT, Kelley MC (2011). RAF inhibitor resistance is mediated by dimerization of aberrantly spliced BRAF(V600E). Nature.

[20] Wilson TR, Fridlyand J, Yan Y, Penuel E, Burton L, Chan E, Peng J, Lin E, Wang Y, Sosman J, Ribas A, Li J, Moffat J, Sutherlin DP, Koeppen H, Merchant M (2012). Widespread potential for growth-factor-driven resistance to anticancer kinase inhibitors. Nature.

[21] Belousov VV, Fradkov AF, Lukyanov KA, Staroverov DB, Shakhbazov KS, Terskikh AV, Lukyanov S (2006). Genetically encoded fluorescent indicator for intracellular hydrogen peroxide. Nat. Methods.

[22] O'Day SJ, Eggermont AMM, Chiarion-Sileni V, Kefford R, Grob JJ, Mortier L, Robert C, Schachter J, Testori A, Mackiewicz J, Friedlander P, Garbe C, Ugurel S, Collichio F, Guo W, Lufkin J (2013). Final Results of Phase III SYMMETRY Study: Randomized, Double-Blind Trial of Elesclomol Plus Paclitaxel Versus Paclitaxel Alone As Treatment for Chemotherapy-Naive Patients With Advanced Melanoma. Journal of Clinical Oncology.

[23] Blackman RK, Cheung-Ong K, Gebbia M, Proia DA, He S, Kepros J, Jonneaux A, Marchetti P, Kluza J, Rao PE, Wada Y, Giaever G, Nislow C (2012). Mitochondrial electron transport is the cellular target of the oncology drug elesclomol. PLoS ONE.

[24] Scarpulla RC, Vega RB, Kelly DP (2012). Transcriptional integration of mitochondrial biogenesis. Trends in Endocrinology & Metabolism.

[25] Vercauteren K, Pasko RA, Gleyzer N, Marino VM, Scarpulla RC (2006). PGC-1-related coactivator: immediate early expression and characterization of a CREB/NRF-1 binding domain associated with cytochrome c promoter occupancy and respiratory growth. Mol Cell Biol.

[26] Gottschalk S, Anderson N, Hainz C, Eckhardt SG, Serkova NJ (2004). Imatinib (STI571)-mediated changes in glucose metabolism in human leukemia BCR-ABL-positive cells. Clinical Cancer Research.

[27] Roesch A, Vultur A, Bogeski I, Wang H, Zimmermann KM, Speicher D, Körbel C, Laschke MW, Gimotty PA, Philipp SE, Krause E, Pätzold S, Villanueva J, Krepler C, Fukunaga-Kalabis M, Hoth M (2013). Overcoming Intrinsic Multidrug Resistance in Melanoma by Blocking the Mitochondrial Respiratory Chain of Slow-Cycling JARID1B(high) Cells. Cancer Cell.

[28] Singhal R, Kandel ES (2012). The response to PAK1 inhibitor IPA3 distinguishes between cancer cells with mutations in BRAF and Ras oncogenes. Oncotarget.

[29] Sotgia F, Martinez-Outschoorn UE, Lisanti MP (2013). Cancer Metabolism: New Validated Targets for Drug Discovery. Oncotarget.

[30] Pelicano H, Carney DA, Huang P (2004). ROS stress in cancer cells and therapeutic implications. Drug Resist Updat.

[31] Beloueche-Babari M, Arunan V, Jackson LE, Perusinghe N, Sharp SY, Workman P, Leach MO (2010). Modulation of melanoma cell phospholipid metabolism in response to heat shock protein 90 inhibition. Oncotarget.

[32] Beck D, Niessner H, Smalley KSM, Flaherty K, Paraiso KHT, Busch C, Sinnberg T, Vasseur S, Iovanna JL, Drießen S, Stork B, Wesselborg S, Schaller M, Biedermann T, Bauer J, Lasithiotakis K (2013). Vemurafenib potently induces endoplasmic reticulum stress-mediated apoptosis in BRAFV600E melanoma cells. Sci Signal.

[33] Paraiso KHT, Haarberg HE, Wood E, Rebecca VW, Chen YA, Xiang Y, Ribas A, Lo RS, Weber JS, Sondak VK, John JK, Sarnaik AA, Koomen JM, Smalley KSM (2012). The HSP90 inhibitor XL888 overcomes BRAF inhibitor resistance mediated through diverse mechanisms. Clinical Cancer Research.

[34] Bonet C, Giuliano S, Ohanna M, Bille K, Allegra M, Lacour J-P, Bahadoran P, Rocchi S, Ballotti R, Bertolotto C (2012). Aurora B is regulated by the mitogen-activated protein kinase/extracellular signal-regulated kinase (MAPK/ERK) signaling pathway and is a valuable potential target in melanoma cells. J Biol Chem.

[35] Kluza J, Jendoubi M, Ballot C, Dammak A, Jonneaux A, Idziorek T, Joha S, Dauphin V, Malet-Martino M, Balayssac S, Maboudou P, Briand G, Formstecher P, Quesnel B, Marchetti P (2011). Exploiting mitochondrial dysfunction for effective elimination of imatinib-resistant leukemic cells. PLoS ONE.

[36] McMenamin ME, Himmelfarb J, Nolin TD (2009). Simultaneous analysis of multiple aminothiols in human plasma by high performance liquid chromatography with fluorescence detection. J. Chromatogr. B Analyt. Technol. Biomed. Life Sci.

[37] Garçon G, Leleu B, Marez T, Zerimech F, Haguenoer J-M, Furon D, Shirali P (2007). Biomonitoring of the adverse effects induced by the chronic exposure to lead and cadmium on kidney function: usefulness of alpha-glutathione S-transferase. Sci. Total Environ.

