# Supplementary material for: Mitochondrial oxidative stress is the achille's heel of melanoma cells resistant to Braf-mutant inhibitor
Source: Oncotarget. 2013 Oct 6;4(11):1986–98. doi: 10.18632/oncotarget.1420 (PMC3875764; doi:10.18632/oncotarget.1420)
Supplement: Supplementary file 1 [file oncotarget-04-1986-s001.pdf]

Mitochondrial oxidative stress is the achille's heel of melanoma cells resistant to Braf-mutant inhibitor - Corazao-Rozas et al

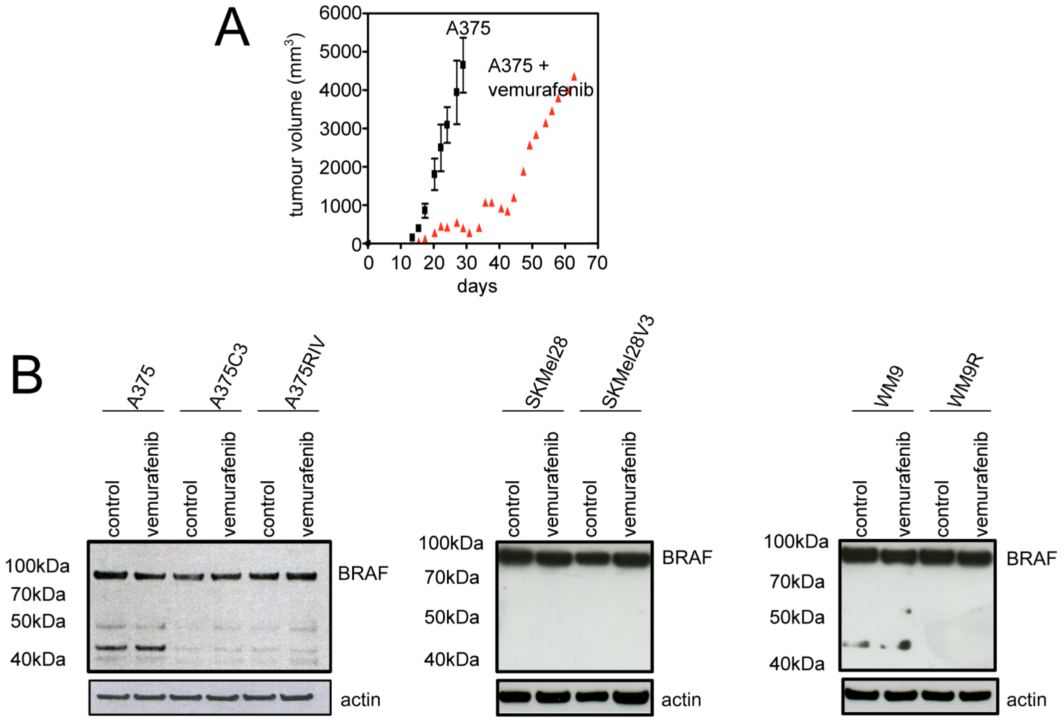

FigS1: (A) Derivation of a variant of A375 (A375RIV) with acquired in vivo resistance to vemurafenib (red). A375RIV cells resistant to the antiproliferative effect of vemurafenib were isolated by continuously treating A375 tumor bearing mice. Tumor growth was measured on the indicated days. After initial regression, tumor growth resumed in the presence of vemurafenib treatment. The persistent tumour was collected and adapted to tissue culture; (B) BRAF expression by immunoblot analysis in human melanoma cells exposed to 3 µM vemurafenib for 6 h. Actin was used as loading control.

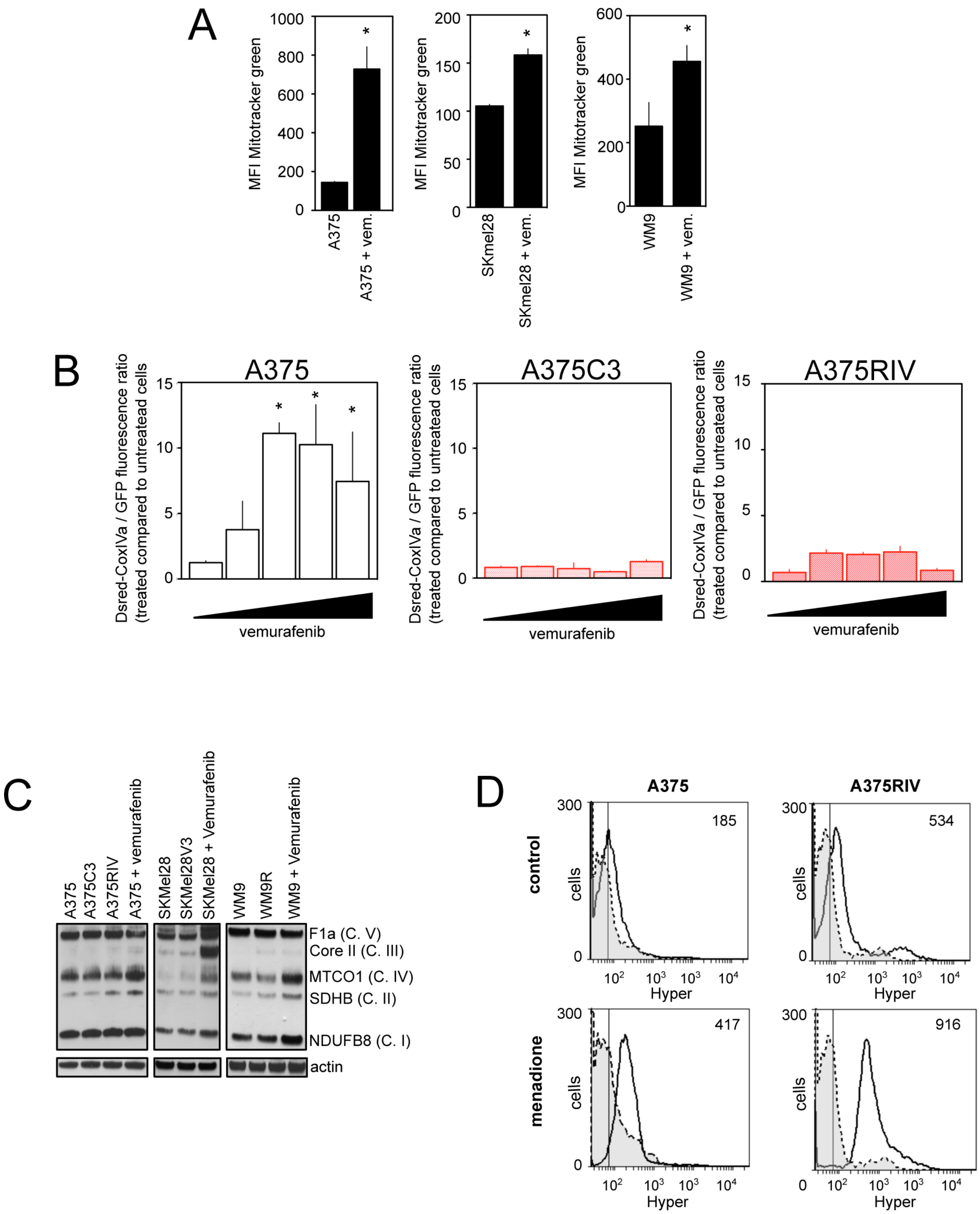

FigS2: (A) Mitochondrial mass in melanoma cells cultured for 72 h in the presence of 3 µM vemurafenib was determined by Mitotracker Green staining and flow cytometry. (Data indicate means of the MFI from three different experiments performed in duplicates ; (B) Cells were transiently co-transfected with DsRed-CoxIVa and GFP then treated with increasing doses of vemurafenib. Ratios of the fluorescence intensities (DsRed to GFP) were plotted. (mean+/-SD, n=4, *p<0.05); (C) Immunoblot analysis of the mitochondrial chain complex proteins : NDUFB8 (at Complex I), SDHB (at complex II), Core 2 protein (at complex III), MTCo (at complex IV) and FI1a protein (at complex V) in cells treated sa in A. Actin was used as a loading control ; (D) Cytometric analysis of mitochondrial ROS production in A375 and A375RIV expressing specific H202 sensor in mitochondria (HyperMito). Cells were kept untreated (control) or treated with the mitochondrial redox cycling promoter menadione (300 µM, 2 h) as positive control of mitochondrial ROS generation. Dashed lines : untransfected cells.

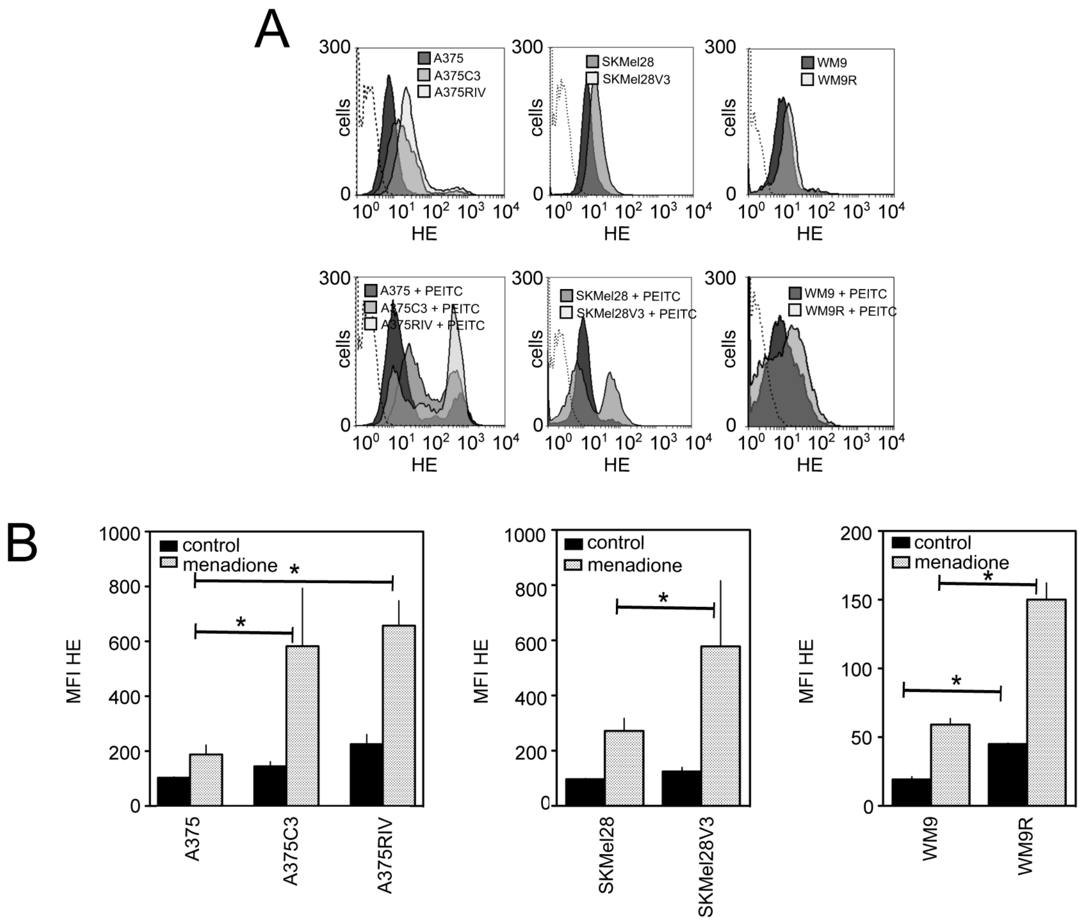

FigS3: (A) To measure ROS generation, melanoma cell lines treated with PEITC (A375, A375C3, A375RIV : 50 µM, for 18h ; SkMel28 and SkMel28V3 :10µM for 6h, WM9 and WM9R : 25µM for 18h). Representative histograms of HE fluorescence from one of three independent experiments are shown ; (B) ROS generation (detected as in A) in melanoma cell lines after menadione exposure. Data are means +/- SD of three independent experiments.
