# Supplementary material for: Mitochondrial oxidative stress is the achille's heel of melanoma cells resistant to Braf-mutant inhibitor
Source: Oncotarget. 2013 Oct 6;4(11):1986–98. doi: 10.18632/oncotarget.1420 (PMC3875764; doi:10.18632/oncotarget.1420)
Supplement: Supplementary file 2 [file oncotarget-04-1986-s002.docx]

Table S1 : Mutational analysis of NRAS, BRAF, and MEK by direct PCR product sequencing

| Sample | Chromosome | Target ID | Hotspot ID | Reference | variation | A  read | C Read | G  read | T  read | % mutant |
| --- | --- | --- | --- | --- | --- | --- | --- | --- | --- | --- |
| A375 | Chr1 | nRAS | Q61K | G | G/T | 7 | 43 | 9272 | 0 | 0% |
| A375C3 | Chr1 | nRAS | Q61K | G | G/T | 10 | 46 | 8832 | 0 | 0% |
| A375RIV | Chr1 | nRAS | Q61K | G | G/T | 5 | 31 | 10225 | 0 | 0% |
| SKMel28 | Chr1 | nRAS | Q61K | G | G/T | 3 | 49 | 10307 | 1 | 0.01% |
| SKMel28V3 | Chr1 | nRAS | Q61K | G | G/T | 5 | 38 | 9382 | 1 | 0.01% |

| Sample | Chromosome | Target ID | Hotspot ID | Reference | variation | A  read | C Read | G  read | T  read | % mutant |
| --- | --- | --- | --- | --- | --- | --- | --- | --- | --- | --- |
| A375 | Chr12 | KRAS | K117N | T | T/C | 0 | 5 | 1 | 7192 | 0.7% |
| A375C3 | Chr12 | KRAS | K117N | T | T/C | 0 | 7 | 0 | 6324 | 0.11% |
| A375RIV | Chr12 | KRAS | K117N | T | T/C | 0 | 5 | 0 | 7115 | 0.07% |
| SKMel28 | Chr12 | KRAS | K117N | T | T/C | 0 | 7 | 0 | 7261 | 0.01% |
| SKMel28V3 | Chr12 | KRAS | K117N | T | T/C | 0 | 11 | 0 | 7649 | 0.14% |

| Sample | Chromosome | Target ID | Hotspot ID | Reference | variation | A  read | C Read | G  read | T  read | % mutant |
| --- | --- | --- | --- | --- | --- | --- | --- | --- | --- | --- |
| A375 | Chr15 | MAP2K1 | C121S | G | G/C | 4 | 0 | 7676 | 0 | 0% |
| A375C3 | Chr15 | MAP2K1 | C121S | G | G/C | 10 | 0 | 8357 | 0 | 0% |
| A375RIV | Chr15 | MAP2K1 | C121S | G | G/C | 8 | 0 | 9213 | 0 | 0% |
| SKMel28 | Chr15 | MAP2K1 | C121S | G | G/C | 7 | 0 | 7735 | 0 | 0% |
| SKMel28V3 | Chr15 | MAP2K1 | C121S | G | G/C | 8 | 0 | 8704 | 0 | 0% |
